# Effect of chronic ethanol consumption in rhesus macaques on the nucleus accumbens core transcriptome

**DOI:** 10.1111/adb.13021

**Published:** 2021-05-04

**Authors:** Nicole Walter, Rita Cervera-Juanes, Christina Zheng, Priscila Darakjian, Denesa Lockwood, Verginia Cuzon-Carlson, Karina Ray, Suzanne Fei, Don Conrad, Robert Searles, Kathleen Grant, Robert Hitzemann

**Affiliations:** 1 Division of Neuroscience, Oregon National Primate Research Center, Oregon Health & Science University, Portland, Oregon, USA; 2 Division of Genetics, Oregon National Primate Research Center, Oregon Health & Science University, Portland, Oregon, USA; 3 Knight Cancer Institute, Oregon Health & Science University, Portland, Oregon, USA; 4 Department of Behavioral Neuroscience, Oregon Health & Science University, Portland, Oregon, USA; 5 Integrated Genomics Laboratory, Oregon Health & Science University, Portland, Oregon, USA

**Keywords:** nonhuman primate, chronic ethanol consumption, nucleus accumbens core

## Abstract

The nucleus accumbens core (NAcc) has been repeatedly demonstrated to be a key component of the circuitry associated with excessive ethanol consumption. Previous studies have illustrated that in a nonhuman primate (NHP) model of chronic ethanol consumption, there is significant epigenetic remodeling of the NAcc. In the current study, RNA-Seq was used to examine genome-wide gene expression in eight each of control, low/binge (LD*), and high/very high (HD*) rhesus macaque drinkers. Using an FDR < 0.05, zero genes were significantly differentially expressed (DE) between LD* and controls, six genes between HD* and LD*, and 734 genes between HD* and controls. Focusing on HD* versus control DE genes, the upregulated genes (*N* = 366) were enriched in genes with annotations associated with signal recognition particle (SRP)-dependent co-translational protein targeting to membrane (FDR < 3 × 10^−59^), structural constituent of ribosome (FDR < 3 × 10^−47^), and ribosomal subunit (FDR < 5 × 10^−48^). Downregulated genes (*N* = 363) were enriched in annotations associated with behavior (FDR < 2 × 10^−4^), membrane organization (FDR < 1 × 10^−4^), inorganic cation transmembrane transporter activity (FDR < 2 × 10^−3^), synapse part (FDR < 4 × 10^−10^), glutamatergic synapse (FDR < 1 × 10^−6^), and GABAergic synapse (FDR < 6 × 10^−4^). Ingenuity Pathway Analysis (IPA) revealed that EIF2 signaling and mTOR pathways were significantly upregulated in HD* animals (FDR < 3 × 10^−33^ and <2 × 10^−16^, respectively). Overall, the data supported our working hypothesis; excessive consumption would be associated with transcriptional differences in GABA/glutamate-related genes.

## 1 | INTRODUCTION

For nearly 40 years,^1,2^ it has been recognized that the nucleus accumbens has a pivotal role in the mechanisms of action associated with alcohol and drugs of abuse,^3–7^ in part due to its implication in the control of motivated behaviors by discrete cues.^8^ Subsequent studies revealed that the nucleus accumbens core (NAcc) and shell (NAcs) have differential roles in the regulation of addictive behavior (see Di Chiara^9^ and references therein). In the current study, the focus is on the NAcc, as it selects and integrates the most relevant information from limbic and cortical efferents to mediate behavior, such as alcohol intake. The NAcc has been implicated in all aspects of alcohol addiction, including dependence, tolerance, craving, and withdrawal.^10–14^

The development and characterization of a nonhuman primate (NHP) model of ethanol consumption^15,16^ has proven to be key for understanding how the brain transcriptome and epigenome are independently linked to a wide range of alcohol consumption histories (see, e.g., literature^1,2,17^). In this animal model, macaques self-select into four distinct, stable drinking categories characterized over 12 months of daily access.^16^ These categorical levels of alcohol intakes are based on the quantity/frequency of consumption and are nominally termed low (LD), binge (BD), heavy (HD), and very heavy (VHD) drinking.^15,18^ Using this model,^1,2^ we have identified epigenetic signals in the NAcc that differentiate alcohol naïve from low/binge from heavy/very heavy alcohol drinking rhesus macaques. Focusing on spatially correlated clusters of differentially methylated CpG sites (differential methylated regions [DMRs]) between the low/binge and heavy/very heavy drinking monkeys, 17 significant DMRs were identified that were significantly correlated with average daily alcohol intake.^1^ Eight of these DMRs mapped to regions near genes are associated with synaptic plasticity; three of the synaptic genes—*LRP5*, *GPR39*, and *JAKMIP1*—revealed significant correlations between DMR and whole-gene or alternative transcript expression (as evaluated by real-time PCR), supporting a functional role of DNA methylation in regulating gene and alternative transcript variant expression. Furthermore, the coordinated changes in DNA methylation and gene expression suggested a bias in the excitatory and inhibitory balance in the NAcc with chronic heavy alcohol use. Following on this hypothesis, and focusing on *GPR39*, a gene encoding the zinc-sensing G protein-coupled receptor 39 previously reported to modulate glutamate and GABA signaling in several brain regions (see references^19^), Cuzon Carlson et al.^19^ found that the GPR39 agonist TC-G 1008 reduced ethanol intake in mice (two-bottle choice [2BC]) without affecting total fluid intake, locomotor activity, or saccharin preference. Furthermore, repeated doses of the agonist prevented ethanol escalation in an intermittent access to 2BC paradigm. This effect was reversible, as ethanol intake increased when the agonist was no longer given. Furthermore, in synaptic recordings of the NAcc, the agonist shifted neurotransmission balance toward excitation, supporting an important role of glutamate signaling in the NAcc in mediating ethanol intake.

The current study was undertaken to expand on these observations that transcriptome changes identified in the NAcc of rhesus macaques underlie excessive ethanol consumption. Genome-wide RNA-Seq was used to examine the NAcc transcriptome in control, low/binge drinking, and heavy/very heavy drinking animals. A key goal of the study was to determine if, as predicted and suggested by the epigenetic studies, excessive chronic ethanol consumption involves genes enriched in annotations associated with synaptic plasticity and specifically glutamate and GABA signaling.

## 2 | METHODS AND MATERIALS

### 2.1 | Animals

NAcc samples were obtained from four cohorts (Cohorts 4, 5, 7a, and 7b) of unrelated adult male rhesus macaques (*Macaca mulatta*) through the Monkey Alcohol Tissue Research Resource (MATRR^20^). The cohort associated with an individual animal is found in Table S1. The animals used here are the same as used previously^1,2^ but with some additions to balance group size. Animals were individually housed in quadrant cages (0.8 × 0.8 × 0.9 m) with constant temperature (20–22°C), humidity (65%), and an 11-h light cycle. Animals had visual, auditory, and olfactory contact with other animals within the same cohort. All animals were maintained on positive caloric and fluid balance, and body weights were recorded weekly. All procedures were conducted in accordance with the Guide for the Care and Use of Laboratory Animals and the NIH guidelines for the care and use of laboratory animal resources and approved by the Oregon National Primate Research Center IACUC. Detailed drinking data and additional data and tissue resources are available for the four cohorts from MATRR (www.matrr.com).^20^

### 2.2 | Ethanol subjects

Monkeys were trained to use operant drinking panels to obtain all fluids (water and/or ethanol) and meals. Once trained, they underwent 4 months of schedule-induced polydipsia (SIP) to induce ethanol self-administration in daily sessions.^16^ SIP is an effective protocol to establish voluntary ethanol consumption through the use of interval schedules for food delivery. Briefly, banana-flavored food pellets (1 g) were delivered every 300 s until the preset volume of fluid was consumed. After SIP of water only, monkeys were induced to increasing volumes of 4% (w/v) ethanol in 30-day increments equivalent to 0.5, 1.0, and 1.5 g/kg/day, respectively. Following induction, “open access” (or 22 h/day concurrent access to ethanol on one spout and water on the other spout) began. The subjects received three meals (approximately 2 h apart) of banana pellets each session (day), with the first meal available at the session start. Each daily session of 22 h began in the late morning and ended early morning the following day. Ethanol was removed for 2 h each day for cage cleaning, blood draws, and other routine procedures. During the 2 h of ethanol removal, monkeys were pair-housed by removing a barrier between adjacent cages. The lighting schedule in the room was 11-h light, 13-h dark; the lights went off 7 h after the start of the open-access session. The details of the open-access protocol are discussed in Grant et al.^16^ as are the methods for BEC determination. Ethanol intake was recorded daily with a 0.5-s resolution. For analysis purposes, the light and binge drinkers (*N* = 8) were combined into a group hereafter denoted as LD*; the heavy drinkers and very heavy drinkers were combined into a group hereafter denoted as HD*.

### 2.3 | Control subjects

Control subjects were housed in the same room as the ethanol drinking subjects of the same cohort and participated in all experimental manipulations. For the controls, SIP and self-administration conditions were identical, with the exception that both spouts dispensed water. A maltose dextrin solution (10% in water) was given to the controls to calorically match the drinkers and controls. The dextrin solution was given at the beginning of each daily session by attaching a bottle to the front of the housing cage.

### 2.4 | Tissue collection

After the 12-month open-access period, and without imposed abstinence, a previously described, detailed necropsy protocol^21^ was used to systematically collect tissues from all subjects. Briefly, monkeys were anesthetized with ketamine (10 mg kg^−1^), maintained on isoflurane, and perfused with ice-cold oxygenated monkey perfusion solution (containing [in mM] 124 NaCl, 23 NaHCO3, 3 NaH2PO4, 5 KCl, 2 MgSO4, 10 D-glucose, 2 CaCl2). Anesthesia was maintained for 30–60 min prior to perfusion. Animals could consume ethanol for up to 4 h prior to sacrifice; blood ethanol concentrations (BECs) at sacrifice were trace to no ethanol detected. Brains were quickly removed and sectioned along the coronal plane using a brain matrix.^21^ The block containing the NAcc was initially selected by each individual’s magnetic resonance imaging and verified using visible landmarks. In macaques, the NAcc is ~2 × 2 mm and extends ~3 mm rostral/caudal.^21^ The core is differentiated from the shell based on visible landmarks such as the curvature of the internal capsule in which the area just ventral to its end is the NAcc. From the frozen 4-mm coronal brain block maintained on dry ice, a small circular dissection of ~1 mm^3^ was made, taking care to not collect white matter from the tract (dorsal to the core). This relatively small dissection avoids the NAcs and yields enough tissue for nucleic acid isolation. RNA was extracted from the NAcc using the All Prep DNA/RNA/microRNA Universal Kit (QIAGEN Sciences, Germantown, MD, USA) following the manufacturer’s recommendations.

### 2.5 | RNA-Seq

Libraries with strand orientation were prepared using the TruSeq RNA Sample Preparation Kit (Illumina, San Diego CA, USA). Libraries were sequenced according to specification on a HiSeq 2500 (Illumina) at the Oregon Health and Science University Massively Parallel Sequencing Shared Resource. PolyA selected libraries were multiplexed three per lane, yielding an average of 76 million total paired-end reads per sample. FastQC (Babraham Bioinformatics) was used for quality checks on the raw sequence data. Reads were aligned to MacaM genome assembly using STAR Version 2.5.2b^22^ with default parameters except for the following: outFilterMismatchNmax = 2 and outFilterMultimapNmax = 1. Using HTSeq Version 0.6.1p1^23^ and the MacaM_Rhesus_Genome_Annotation_v7.8.1 GTF annotation file, read counts were summarized at the gene level. On average, 70% of reads are uniquely mapped to the MacaM genome. Generalized linear models, using DESeq2,^24^ were used to detect differential expression between high drinkers and controls (HD* vs. controls), low drinkers and controls (LD* vs. Control), and high drinkers and low drinkers (HD* vs. LD*). A secondary analysis contrasted the HD and VHD drinkers (*N* = 4 each) within the HD* group; details are found in Data S1. The RNA-Seq data are available through NCBI GEO database (accession # GSE144783).

### 2.6 | Gene ontology enrichment

Gene ontology (GO) enrichment was conducted on the ethanol-affected differentially regulated genes using GOrilla^25^ with human annotations; transcripts meeting the threshold of one transcript per million (TPM) were used as the background reference. The significance level was set at FDR (*q*) < 0.05.

### 2.7 | Ingenuity Pathway Analysis

Pathway analyses^26^ were performed in Ingenuity Pathway Analysis (IPA) (QIAGEN Inc., https://www.qiagenbioinformatics.com/products/ingenuitypathwayanalysis). Pathway analysis for HD* versus controls was performed at FDR < 0.05.

### 2.8 | Quantitative polymerase chain reaction

RNA extracted from the same NAcc tissues as used for the RNA-Seq analysis was used for real-time quantitative reverse transcriptase PCR (RT-PCR) analysis. The Fluidigm Reverse Transcription Master Mix (Fluidigm, San Francisco, CA, USA) was used to reverse-transcribe 25 ng of each RNA sample following the manufacturer’s instructions. Next, the complementary DNA was pre-amplified, and unincorporated primers were removed following the manufacturer’s instructions. The reactions were diluted (10×) with 43 μL of TE buffer (Teknova, Hollister, CA, USA). Quantitative polymerase chain reaction (qPCR) was performed using the BioMark HD System and the 96.96 GE Dynamic Arrays (Fluidigm) in triplicate assays. The Fluidigm sample premix and the assay premix were prepared following the manufacturer’s instructions. The samples and reagents were mixed using the Nanoflex IFC controller (Fluidigm). Thermal qPCR conditions were as follows: 95°C for 60 s, 35 cycles of 95°C for 5 s, and 60°C for 20 s. Data were processed by automatic threshold for each assay, with derivative baseline correction using the BioMark Real-Time PCR Analysis Software 3.1.2 (Fluidigm). The quality threshold was set at the default of 0.65. The mRNA expression levels were normalized as previously described^1,2^ except for using cytochrome c1 as the constitutive gene (*CYC1*). Among eight potential constitutive genes (*B-ACTIN*, *PGK1*, *TOP1*, *ACTG1*, *RPL13*, *UBE2D2*, *EIF4A2*, and *CYC1*), we selected *CYC1* for housekeeping as determined by ReFinder.^27^ Three sets of triplicate assays were performed for each sample. The primer sequences are listed in Table S6.

## 3 | RESULTS

### 3.1 | Voluntary ethanol consumption

NAcc samples were obtained from eight controls, eight low/binge drinkers, and eight heavy/very heavy drinkers. The drinking categories are based on data-driven definitions.^15^ For simplicity, we refer to the two drinking groups as LD* and HD*. Average daily consumptions in the LD* versus HD* were 1.58 ± 0.19 versus 3.14 ± 0.12 g/kg (*t*-test—*p* < 5 × 10^−6^) (see Table S1). BECs were measured approximately every 5 days during the 12-month open-access period at 7 h into the daily 22 h of open access. Due to the timing of the blood sampling, the BECs do not necessarily reflect the total daily intake, but rather the intake pattern over the first 7 h of session access. Average BECs in LD* versus HD* were 42 versus 97 mg/dL (*p* <9 × 10^−5^). All of the animals were between 5 and 8 years of age, except for two older LD* animals. Age was not a significant covariate in the analyses. Box plots of the BEC data across groups are also found in Supplemental Table S1. Additional details on the animals are available at the MATRR^20^ and can be accessed using the MATRR ID.

### 3.2 | Differential gene expression: RNA-Seq

Thirteen thousand six hundred thirty-seven genes met the threshold of one TPM (reads) for inclusion in the data analysis. These genes and their expression levels in the three groups are listed in Table S2. These genes were used as the reference set for annotation (see below). It should be noted that the animals used were expressly chosen to be genetically unrelated and principal component analysis (PCA) revealed that differences among animals were greater than the differences among groups (data not shown).

Generalized linear models, using DESeq2,^24^ were used to detect differential expression among the three groups. No genes were significant with an FDR < 0.05 for LD* versus controls, six genes were significant with an FDR < 0.05 for HD* versus LD*, and 734 genes were found to be significant with an FDR < 0.05 for HD* versus controls. Further analyses focused solely on the genes that differentiated the HD versus controls.

Focusing on the HD* versus controls contrast and meeting the threshold of FDR < 0.05, 366 genes were upregulated in HD* compared with controls, and 368 were downregulated (Table S3). Three hundred sixty each of the upregulated and downregulated genes were annotated; 13,294 genes in the reference set were annotated. Using the GOrilla algorithm,^25^ the upregulated genes were highly enriched in genes associated with ribosomal function (Table S4); key annotations included signal recognition particle (SRP)-dependent co-translational protein targeting to membrane (FDR < 3 × 10^−59^), structural constituent of ribosome (FDR < 3 × 10^−47^), and ribosomal subunit (FDR < 5 × 10^−48^). Enrichr^28,29^ was queried to search for common transcription factors (TFs). Significantly associated TFs included *MYC* (FDR < 7 × 10^−20^), *KAT2A* (FDR < 1 × 10^−10^), and *TAF7* (FDR < 9 × 10^−14^); none of these TFs were differentially expressed. Focusing on TF protein–protein interactions (PPIs) significant associations were detected for *ILF3* (FDR < 7 × 10^−19^), *ILF2* (FDR < 4 × 10^−13^), and *RAD21* (FDR < 5 × 10^−14^). Of these, the expression of *ILF2* showed a trend to a significant increase (Table S2; FDR < 0.09). The upregulated genes were significantly enriched in miRNA binding sites for miR-652–3p (FDR < 5 × 10^−5^), miR-124–3p (FDR < 5 × 10^−5^), and miR-100–5p (FDR < 2 × 10^−3^).

**FIGURE 1:**
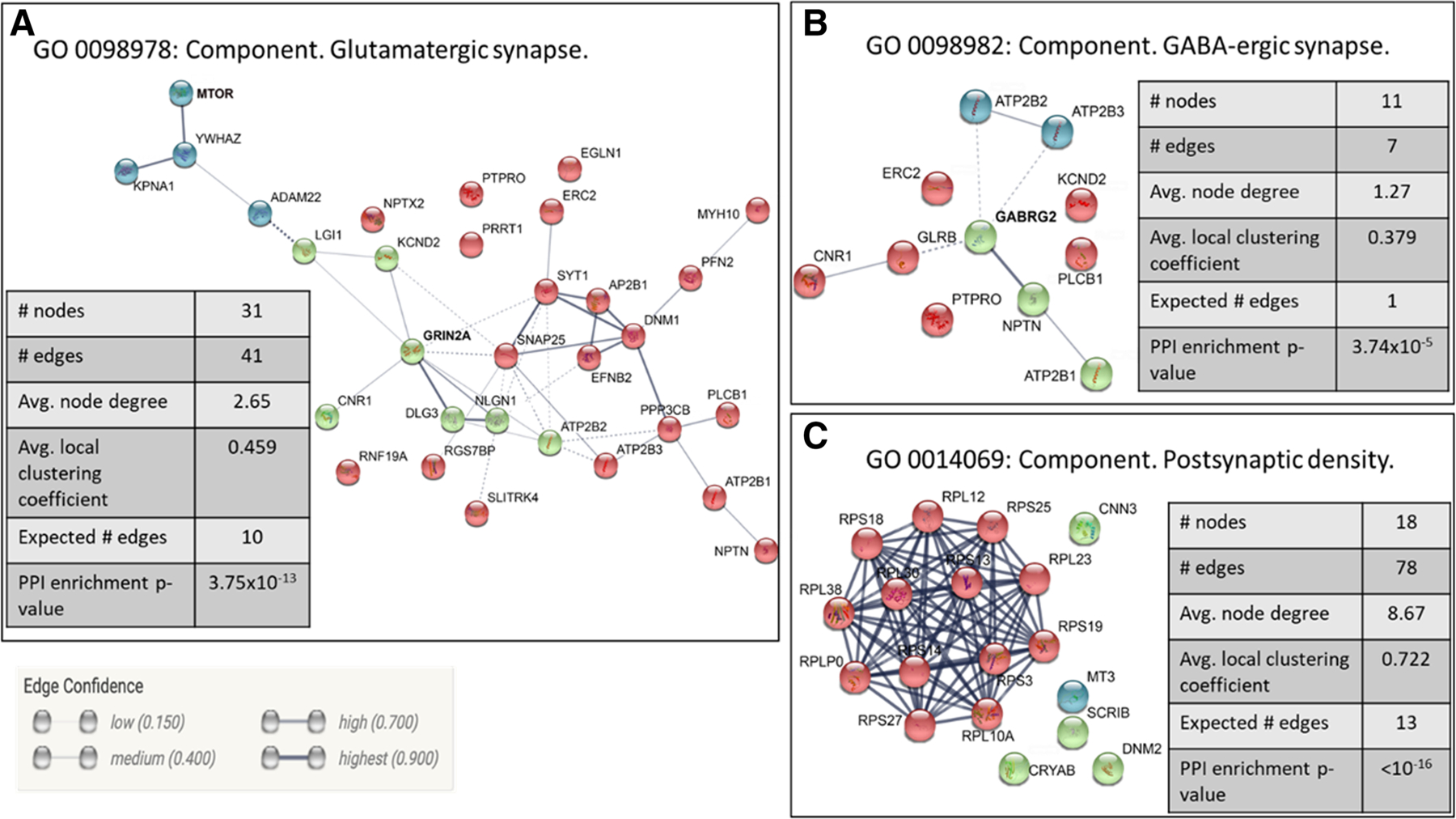
Network representation of three GO-enriched pathways. Genes in (a) and (b) were downregulated while in (c) was upregulated in heavy drinkers as compared with controls. Networks were created using String v11.0. Networks were built using text mining, experiments, databases, and co-expression datasets. The nodes represent proteins encoded by the differentially expressed genes. Edges represent protein–protein interactions. The edge confidence is represented by the thickness of the edge (as shown in the edge confidence description in the figure). Networks are clustered using k-means clustering

Key annotations for the downregulated genes included behavior (FDR < 2 × 10^−4^), membrane organization (FDR < 1 × 10^−4^), inorganic cation transmembrane transporter activity (FDR < 2 × 10^−3^), synapse part (FDR < 4 × 10^−10^), glutamatergic synapse (FDR < 1 × 10^−6^), and GABAergic synapse (FDR < 6 × 10^−4^) (Table S5; Figure 1). The genes associated with the glutamate and GABA annotations are listed in Table 1. Genes that overlap in the two categories are noted in italics and include *ATP2B1*, *ATP3B2*, *ATP2B3*, *CNR1*, *ERC2*, *KCNDS*, *NPTM*, *PLCB1*, and *PTPRO*. Enrichr was queried to search for common TFs. Significantly associated TFs included *UBTF* (FDR < 3 × 10^−12^), the UBTF interaction partner *TAF1* (FDR < 8 × 10^−10^), and *CREB* (FDR < 4 × 10^−7^). None of these TFs showed significant DE. Focusing on TF PPIs significant associations were detected for *XRCC4* (FDR < 3 × 10^−3^), *SMC3* (FDR < 1 × 10^−3^), and *ESR1* (FDR < 2 × 10^−5^). Neither *XRRC4* nor *SMC3* exhibited DE; ESR1 expression, although detectable, was below the threshold for analysis (see above). Differently than for the upregulated genes, the downregulated genes were markedly enriched in miRNA binding sites; these included miR-340–5p (FDR < 8 × 10^−16^), miR-21–5p (FDR < 3 × 10^−14^), and miR-19b-3p (FDR < 4 × 10^−10^).

**FIGURE 2:**
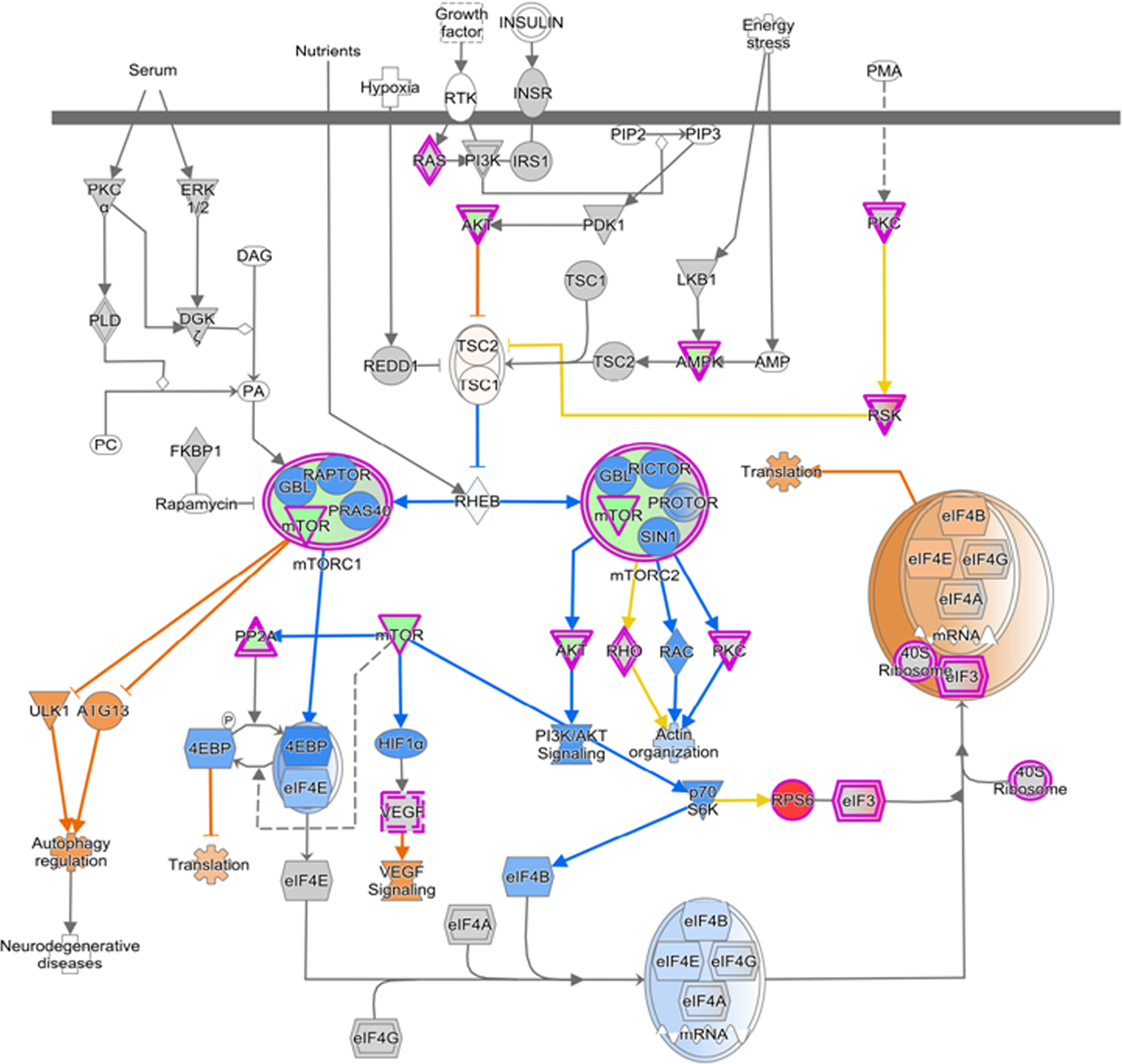
Ingenuity Pathway Analysis observed and predicted impact on mTOR signaling in HD versus control. Red and green indicate observed increased and decreased expression, respectively. Orange and blue indicate predicted activation and inhibition, respectively

IPA was run on the entire set on DE genes using the directionality of gene expression change as an input variable. EIF2 signaling and mTOR pathways were significantly upregulated in HD animals (FDR < 3 × 10^−33^ and <2 × 10^−16^, respectively). Signaling pathways inhibited in HD animals included CREB signaling in neurons (FDR < 9 × 10^−7^; Figure 2), synaptic long-term depression (FDR < 4 × 10^−10^) and potentiation, synaptogenesis (FDR < 4 × 10^−10^), tight junction (FDR < 4 × 10^−10^), endocannabinoid neuronal synapse (FDR < 4 × 10^−10^), opioid signaling (FDR < 4 × 10^−10^), neuregulin signaling (FDR < 4 × 10^−10^), and PPARα/RXRα activation (FDR < 4 × 10^−10^). Common molecules among these pathways include the glutamate receptor subunit genes *GRIA3*, *GRIN2C*, *GRM5*, and *GRM7*.

Although there were only four each HD and VHD animals in the HD* group, it was of interest to determine if the annotations noted above were enriched in both or only one group. Results of this analysis are found in Table S7; analysis details are found in the Supplemental pdf. Six hundred forty-two genes were DE between the HD and VHD animals (FDR < 0.001); 414 genes were more highly expressed in the HD animals, and 228 genes were more highly expressed in the VHD animals. For the HD > VHD genes, the annotations included behavior (FDR < 2 × 10^−11^), chemical synaptic signaling (FDR < 8 × 10^−10^), cation transmembrane transporter activity (FDR < 8 × 10^−8^), synapse (FDR < 8 × 10^−19^), and glutamatergic synapse (FDR < 6 × 10^−14^). TFs significantly associated with this gene grouping included SUZ12 (FDR < 3 × 10^−17^), SMAD4 (FDR < 6 × 10^−8^), GATA1 (FDR < 3 × 10^−3^), NFE2L2 (FDR < 3 × 10^−3^), SOX2 (FDR < 4 × 10^−3^), CREB1 (FDR < 3 × 10^−3^), and EGR1 (FDR < 5 × 10^−3^). Of this group of TFs, only EGR1 was differentially expressed (HD > VHD, FDR < 3 × 10^−4^). Genes in this grouping were markedly enriched in miRNA binding sites that included miR-466b-5p (FDR < 2 × 10^−16^), miR-297a (FDR < 2 × 10^−15^), and miR-467 h (FDR < 4 × 10^−14^).

For the VHD > HD genes, the annotations included SRP-dependent co-translational protein targeting to membrane (FDR < 7 × 10^−17^), translation (FDR < 2 × 10^−10^), structural constituent of ribosome (FDR < 6 × 10^−12^), and ribosomal subunit (FDR < 6 × 10^−11^). TFs significantly associated with this gene grouping included EDF1 (FDR < 1 × 10^−20^), POLR2L (FDR < 5 × 10^−20^), YBX1 (FDR < 6 × 10^−17^), GTF3A (FDR < 5 × 10^−16^), ZMAT2 (FDR < 4 × 10^−16^), POLE4 (FDR < 3 × 10^−15^), and ATF4 (FDR < 3 × 10^−14^). With the exception of EDF1 and ZMAT2, the remaining TFs were significantly (FDR < 0.05) more highly expressed in the VHD group. In contrast to the TFs, there was in the VHD > HD group no significant enrichment in miRNA binding sites.

### 3.3 | Differential gene expression: qPCR

Real-time qPCR was used to validate the expression of a subset of the DE genes. In agreement with the RNA-Seq data, *ARHGEF7* and *GRM7* were downregulated in HD* animals as compared with controls (independent *t*-tests; *p <* 7 × 10^−3^ and *p <* 2 × 10^−2^, respectively). Although the differential expression between controls and HD* animals did not reach significant levels for *GRIA* and *GRM5*, there was a trend toward down-regulation in HD animals (*p<* 9 × 10^−2^ and *p<* 8 × 10^−2^, respectively).

**FIGURE 3:**
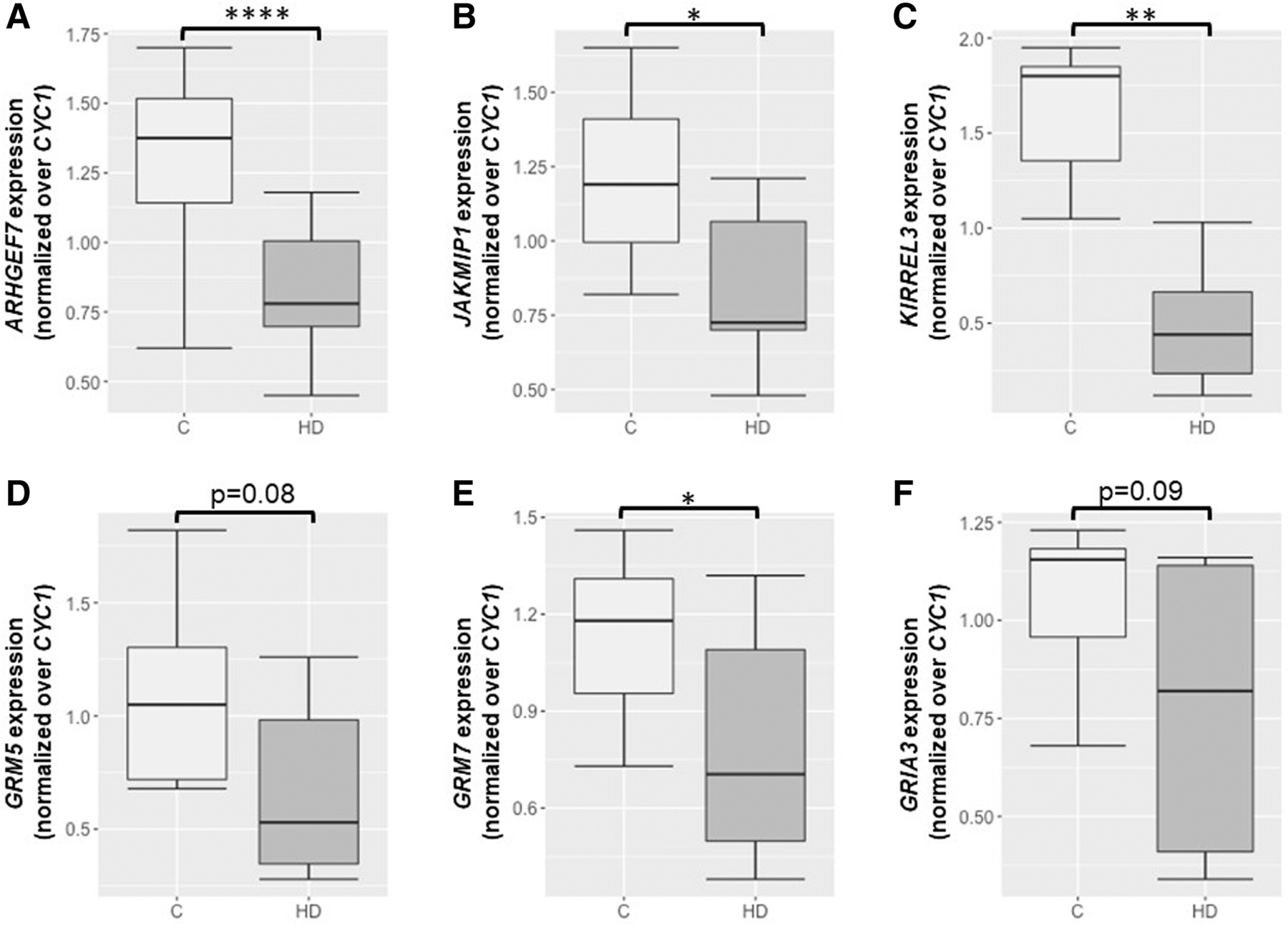
Summary of the gene expression profiles of six genes analyzed by qPCR. The relative expression of ARHGEF7 (a), JAKMIP1 (b), KIRREL3 (c), GRM5 (d), GRM7 (e), and GRIA (f) between controls (^ʹ^C) and heavy/very heavy (HD) drinkers is shown; independent *t*-test with Bonferroni correction for multiple comparisons, *p < 0.05, **p < 0.01, ****p < 0.0001. Error bars are mean +/− SEM

We also investigated the expression levels of two genes, *KIRREL3* and *JAKMIP1*, which were previously identified as differentially methylated between low and heavy/very heavy drinkers.^1,2^ Although these genes did not show significant differential expression after FDR correction in our RNA-Seq analysis (Table S2), they showed a significant downregulation of specific transcript variants (see Figure 3) in HD* animals as compared with controls.

## 4 | DISCUSSION

Iancu et al.^17^ were the first to examine in rhesus macaques and at a genome-wide level with RNA-Seq the relationships between chronic ethanol consumption and the brain transcriptome. Thirty-one animals were entered into the analysis. Daily ethanol consumption ranged from 0.52 to 3.79 g/kg with a relatively normal distribution. Two brain regions were examined: the central nucleus of the amygdala (CeA) and cortical area 32. However, it was the CeA RNA-Seq data that showed the most robust associations with ethanol consumption. The genes positively correlated to consumption were enriched (FDR < 0.05) in annotations associated with the regulation of axon extension, postsynaptic chemical synaptic transmission, and membrane part. The postsynaptic genes included *Gsk3β*, which has been linked to excessive ethanol consumption.^30,31^ These positively correlated genes were significantly enriched in a co-expression network module that was richly annotated with synaptic, neuronal, and membrane GO categories. This module included a number of neurotransmitter receptors (e.g., *Chrm3*, *Chrna4*, *Chrna7*, *Glra2*, *Grm1*, *and Grm2*).

Iancu et al.^17^ also observed that the genes negatively correlated to intake were enriched in GO categories associated with nucleic acid processing and transcription. Previously, it was observed that alcohol-preferring inbred mouse strains and animals selectively bred for high alcohol preference have an increased expression of genes associated with transcription, notably key TFs.^32^ Subsequent studies confirmed the alcohol/transcription interaction.^33–36^ Ponomarev et al.^36^ found, when comparing chronic alcoholics and normal controls, that there was a modest upregulation of CeA ribosomal genes in the alcoholics. The authors suggested that this may be a consequence of DNA hypomethylation (see below for discussion).

From a broad perspective, the data presented in Iancu et al.^17^ and the data presented here are mirror images. We observed that in the NAcc, the genes upregulated in the HD* animals were very strongly associated with translation, whereas the genes downregulated in were enriched in annotations associated with synaptic function, including glutamate and GABA synaptic plasticity. The increased expression of genes associated with translation, including a large number of ribosomal proteins, is consistent with the large number of studies noted above but differs from Iancu et al.^17^ At the moment, we can only speculate as to the reasons for the differences between studies. Perhaps the most logical inference is that the differences reflect regionally specific effects of excessive ethanol consumption on transcription; there is considerable evidence for regionally specific effects.^37^ We report here the mTOR signaling pathway as being activated in the HD* animals. These findings agree with the extensive literature supporting mTOR activation with both alcohol administration and excessive voluntary consumption in the NAc of rodents.^38^ Interestingly, there is complex synergy between mTOR and GABA and glutamatergic neurotransmission.^38,39^

The downregulated genes in the NAcc were enriched in synaptic annotations including a number of GABA, glycine, and glutamate sub-units and receptors: *GABRG2*, *GLYR2*, *GRIA3*, *GRIN2A*, *GRM5*, and *GRM7*. In addition, a cluster of potassium channel proteins were downregulated: *KCND2*, *KCNH1*, *KCNJ9*, and *KCNT1*. These data are consistent with the repeated observation that chronic ethanol consumption is associated with the involvement of GABA, glycine, endocannabinoids, and glutamate systems and associated potassium channels.^1,2,19,40–49^ The transcriptional data cannot inform one as to how chronic ethanol consumption affects the balance between inhibitory and excitatory tones. However, the available data generally support the idea of an impaired balance within key regions of the addiction circuit.^38,50–60^

Bogenpohl et al.^61^ used microarrays to probe differences in the cortical transcriptome of animals in the same cohorts as used in the current study; the number of drinkers was double that used in the current study. We have some concern comparing microarray and RNA-Seq data because of the marked differences in variance structure.^17^ Nonetheless, we compared the genes in Table S3 with those gene probesets in Bogenpohl et al.^16^ that had an ethanol-related hub node score of >2 (Scales 0–3); this list included ~11% of the probesets that passed the threshold for inclusion in the data analysis. One hundred ninety-three of the 734 genes in Table S3 overlapped with Bogenpohl et al.^16^ (see Table S9). The overlap was significant (*p* < 0.0001). Annotations for the overlapping gene set are also found in Table S9. Annotation enrichments were similar to those for the entire set of genes listed in Table S3 and included synapse (FDR < 2 × 10^−4^), translation (FDR < 3 × 10^−5^), and structural constituent of ribosome (FDR < 6 × 10^−6^). Overlapping genes in the synapse category are also found in Table S9. Many of the same genes are noted above and include *GABRG2*, *GRIA3*, *GRM5*, *GRM7*, and *KCND2*. Similar to the core, *GABRG2* and *GRIA3* were downregulated; however and differently, *GRM5*, *GRM7*, and *KCND2* were upregulated in the cortex.

Although the numbers were small, if the broadly defined HD* group is divided into the more specifically defined HD and VHD groups, significant differences between these groups were detected. Synaptic annotations were overrepresented in the HD group, whereas translational annotations were overrepresented in the VHD group. These differences need to be independently replicated. However, the results importantly suggest that the HD and VHD are not simply part of the same continuum. Seemingly, this is an important alert for not only future analyses of macaque data but also data collected in other animal model and human studies.

The mechanisms linking chronic ethanol exposure to changes in the transcriptome are unclear. It is reasonable to assume that some of the differences between the controls and high/very high drinkers are associated with risk alleles. Selective breeding in rodents^62,63^ have clearly demonstrated that risk alleles involve synaptic function and genes associated with glutamate synaptic plasticity. However, some of the differences between the controls and HD animals observed in the current study must be the result of ethanol exposure per se on the epigenome. Cervera-Juanes et al.^1,2^ addressed this issue by searching for differentially methylated signals in the NAcc among LD and HD rhesus macaques. Focusing on the differentially methylated regions (DMRs) between animals, 17 significant DMRs significantly correlated with the dose of alcohol consumed were identified. Eight of the DMRs mapped to regions near genes are associated with synaptic plasticity. In the present study, *ARHGEF7* (TPM: 44, log2 fold change: −0.33, FDR < 0.05) and *NBEA* (TPM: 57, log2 fold change: −0.68. FDR < 0.05) showed significant downregulation in HD animals as compared with control subjects, consistent with being hypermethylated. Two other genes, *KIRREL3* (TPM: 47, log2 fold change: −0.37, FDR < 0.13) and *JAKMIP1* (TPM: 157, log2 fold change: −0.29, FDR < 0.27), were trending in the anticipated direction, that is, down-regulation. Overall, the agreement between Cervera-Juanes et al.^2^ and the current study is reasonable; the probability of detecting two of the hypermethylated genes significantly downregulated is reasonably small (*p* <4 × 10^−4^) and unlikely to be a chance observation. The observation that two additional genes were trending in the right direction adds significance and supports a relationship between DNA methylation in the regulation of expression of these genes. The lack of further overlap between these two datasets may be due to the fact that we are assuming that the DMRs detected by Cervera-Juanes et al.^2^ between LD and HD animals will be similar to those detected between controls and HD animals; this may only be true for a small set of DMRs and genes, as suggested here. In addition, and based on the strong role of DNA methylation in modulating alternative transcript expression in the context of alcohol, we are currently investigating the role of DNA methylation specifically between controls and high/very high drinkers in modulating specific transcript variant expression.

Five genes (*KRAS*, *CACNA1L*, *GRIN2A*, *GRM5*, and *GABRG2*) DE in HD animals versus controls were previously identified in GWAS of AUDs.^64^ Further, 44 DE genes (Table S7) were previously identified in a microarray study of postmortem human nucleus accumbens comparing individuals with AUDs and matched controls.^65^ The common genes were present in co-expression modules specifically enriched for different brain-specific cell types and included both neurons and astrocytes. Neuronal genes included *DLG3*, *GABRG2*, and *SNAP25*; glial genes included *AGT*, *CD63*, and *NPC2*.

Other membrane genes (Table S5) with known relationships to alcoholism and/or alcohol related phenotypes were detected as downregulated in the high/very high drinkers. This list includes *CACNA1C*, *CACNA1L*, *GPR158*, *KCNJ9*, *NOS1*, *PRCKE*, and *SCN3B*; supporting evidence is found in the literature.^66–72^ It is not our intent to describe the relationships between these genes and alcohol phenotypes in any detail. Rather, we argue that it is important to note that this list of genes was largely nominated from rodent and human studies and have now been confirmed in the NHP model.

Walter et al.^73^ have recently examined in cynomolgus macaques the effects of chronic ethanol exposure on the transcriptome using a within-subject design; area 46 was biopsied prior to ethanol exposure and compared with a contralateral sample taken at necropsy. Controls were treated similarly but without ethanol exposure. In the controls, 77 genes were uniquely DE (biopsy vs. necropsy), 99 genes were DE in both controls and drinkers, and 1242 were uniquely DE in the drinkers. About the set unique to the drinkers, we make two observations. First, the upregulated genes (*N* = 567) were significantly enriched in genes associated with receptor function and included *CHRM2*, *DRD5*, *GABRA1*, *GABRA4*, *GABRG2*, *GLRB*, *HTR2A*, *HTR2C*, and *NPYR5*. In comparison with the current study, it is pertinent to note that the GABA receptor subunits in Walter et al.^73^ were upregulated. Although a different species was used in Walter et al.^73^, the data comparison perhaps again points to regionally specific effects. Walter et al.^73^ also noted that among the genes uniquely downregulated in the drinkers, there was a significant enrichment in genes associated with inflammatory function/response. There is now robust evidence of relationships between neuroimmune signaling and AUDs.^74–76^ Neuroinflammatory mechanisms are associated with both the risk for and individual variation in excessive ethanol consumption.^17,77^ The current study suggests that an inflammatory signature, if present in the NAcc, was below our level of detection.

Overall, the data presented here illustrate that in the NAcc there are significant transcriptome differences in rhesus macaques between controls and heavy/very heavy drinking animals. These are some of the first data to examine the transcriptional features associated with heavy/very heavy drinking in an NHP model of excessive ethanol consumption. Our working hypothesis was that excessive consumption would be associated with transcriptional differences in GABA/glutamate-related genes. The data support this hypothesis.

## Supplementary Material

Supplemental tables

## Figures and Tables

**TABLE 1:** Glutamate and GABA Associated Genes Down-regulated in the NAcc

Glutamate	GABA
ADAM22	
AP2B1	
ATP2B1	ATP2B1
ATP2B2	ATP2B2
ATP2B3	ATP2B3
CNR1	CNR1
DLG3	
DNM1	
EFNB2	
EGLN1	
ERC2	ERC2
GRIA3	GABRG2
GRIN2A	GLRB
GRM5	
GRM7	
KCND2	KCND2
KPNA1	
LGI1	
MTOR	
MYH10	
NLGN1	
NPTN	NPTN
NPTX2	
PFN2	
PLCB1	PLCB1
PPP3CB	
PRRT1	
PTPRO	PTPRO
RGS7BP	
RNF19A	
SLITRK4	
SNAP25	
SYT1	
YWHAZ	
